# A Case of Multifactorial Viral Myocarditis

**DOI:** 10.7759/cureus.18950

**Published:** 2021-10-21

**Authors:** Sahil Zaveri, Ariana R Tagliaferri, Sara Woldemariam, Polina Aron, Carlos Palacios, Gabriel Melki, Patrick Michael

**Affiliations:** 1 Internal Medicine, St. Joseph's University Medical Center, Paterson, USA; 2 Gastroenterology, St. Joseph's University Medical Center, Paterson, USA

**Keywords:** coxsackievirus, viral infection, moderna vaccine, myocarditis, covid-19

## Abstract

We present a case of viral myocarditis in the setting of Coxsackievirus and coronavirus disease 2019 (COVID-19) infection. This case is unique as there were two underlying active infections that could have caused the patient’s myocarditis. Though both viruses have been shown to cause myocarditis, it was difficult to differentiate the exact etiology in this particular case. The unique nature of this case presents the opportunity to explore whether further diagnostic workup is warranted.

## Introduction

Myocarditis is an inflammatory condition of the myocardium which can lead to dilated cardiomyopathy [1-3]. Numerous etiologies have been noted and studied, varying from viral (adenovirus, Coxsackie B virus, parvovirus B19, and HIV), parasitic (*Trypanosoma cruzi and* *Toxoplasma gondii*), bacterial (*Borrelia burgdorferi*, *Mycoplasma pneumoniae*, *Corynebacterium diphtheriae*, and *Campylobacter jejuni*), rheumatic fever, drugs (doxorubicin, cocaine, and sulfonamides), and autoimmune (Kawasaki disease, sarcoidosis, systemic lupus erythematosus, polymyositis, and dermatomyositis) [4,5]. The pathophysiology of myocarditis is not well-understood, but there are several theories developed [5,6]. It is thought that acute and chronic damage to the myocardium is due to T lymphocytes and autoreactive antibodies [2]. Studies using animal models have demonstrated direct viral proliferation within myocytes leading to their destruction [4].

The treatment for myocarditis varies on the clinical presentation [2]. If patients have symptoms of acute heart failure, angiotensin-converting enzyme inhibitors, angiotensin receptor blockers, aldosterone antagonists, and β-adrenergic blockers are indicated [2]. Some studies have shown evidence to support the use of immunosuppressants or steroids, depending on the duration of symptoms [2]. In patients with symptoms lasting less than six months, there was no benefit for immunosuppressants or steroids; however, there was a benefit and adequate clinical response if symptoms lasted longer than six months [2].

In this case report, we present a case of myocarditis in a previously young healthy male who recently tested positive for coronavirus disease 2019 (COVID-19), despite completing a full course vaccination series. A study focusing on the safety and efficacy of vaccine administration observed that systemic adverse effects were more common in younger participants who received the vaccination [5-7]. There is significant anecdotal evidence surrounding vaccination reactions; however, this should not deter patients from obtaining the COVID-19 vaccine [5,7]. This report highlights the various etiologies of myocarditis and explores the ways to further differentiate etiologies if more than one virus or cause is likely in a patient with confirmed myocarditis.

## Case presentation

**Figure 1:**
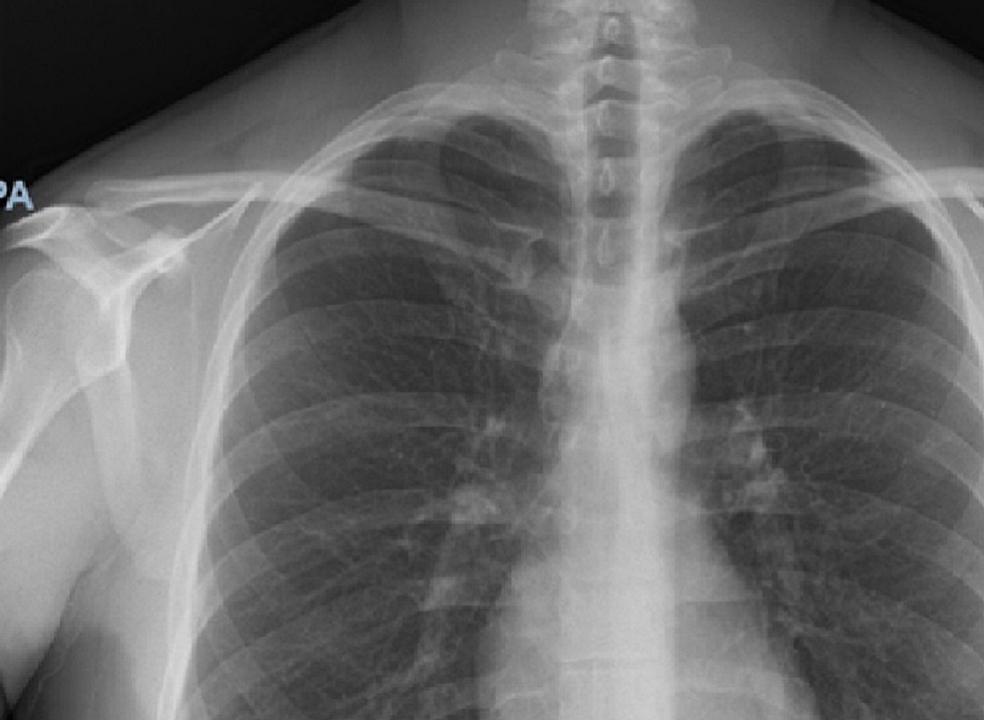
Chest x-ray on admission. There is no focal consolidation or congestive heart failure. There is no pleural effusion, cardio-mediastinal silhouette is not enlarged, and trachea is in the midline. There is no pneumothorax.

**Figure 2:**
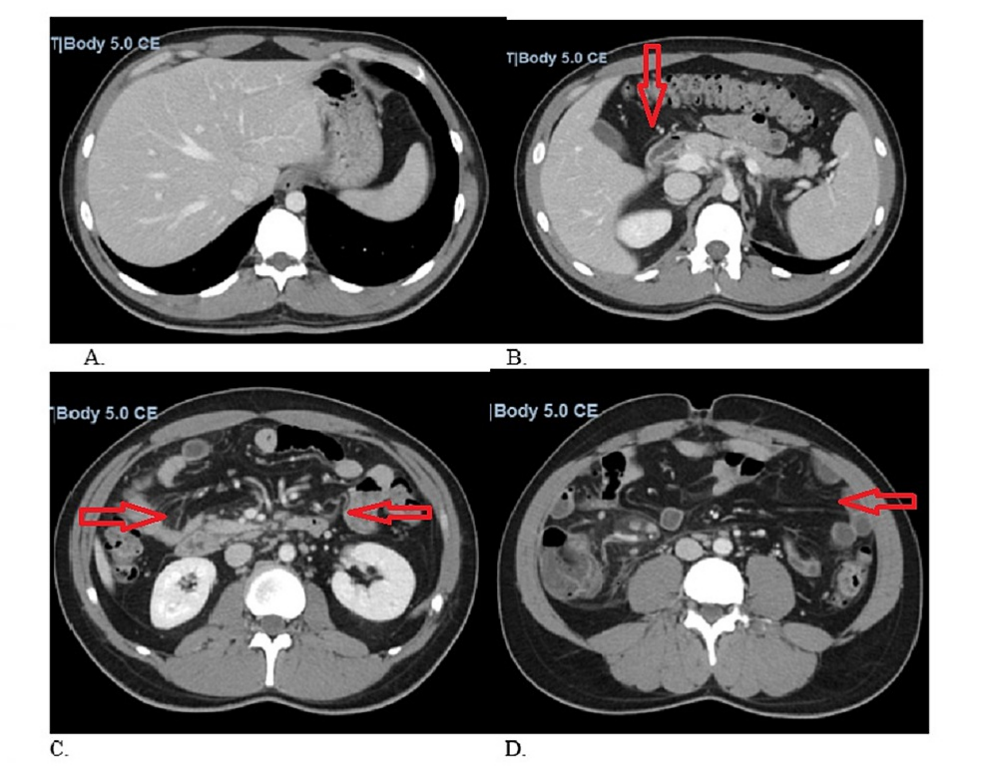
CT of the abdomen and pelvis with intravenous contrast. Axial CT through the abdomen and pelvis with IV contrast. Focal fat along the falciform ligament (A). There is wall thickening of the terminal ileum, cecum, and ascending colon (B). There is mesenteric stranding and lymphadenopathy noted in the right mesentery (C and D). There is no free air, no ascites, and no evidence of gastrointestinal obstruction.

A 32-year-old male with no significant past medical history presented to the emergency department (ED) with generalized fatigue, nausea, abdominal pain, and diarrhea. The patient tested positive for COVID-19 six weeks prior and only suffered anosmia and ageusia, which resolved after his two-week quarantine. Three weeks later, the patient obtained the Moderna vaccine after which he developed diarrhea and abdominal pain prompting his first visit to the ED. At that time, he was discharged home on ciprofloxacin and Flagyl for colitis; however, his symptoms persisted prompting further evaluation. Upon arrival of this current visit, the patient was hypotensive (89/57 mmHg), tachycardic (130 beats per minute), afebrile, and was saturating 89% on room air. Chest x-ray (CXR) in the ED showed no acute cardiopulmonary disease (Figure 1). Labs were remarkable for leukocytosis (15 x 10^3^/mm^3^), elevated high-sensitivity troponin (864 ng/mL), and brain natriuretic peptide (2322 pg/nL). His severe acute respiratory syndrome coronavirus 2 (SARS-CoV-2) PCR test was positive on admission. Electrocardiogram (EKG) showed sinus tachycardia with a ventricular rate of 142 beats per minute, first-degree atrioventricular block, without any ST-segment changes or other abnormalities. Computerized tomography (CT) of the abdomen and pelvis with intravenous contrast showed nonspecific colitis (Figures 2A-2D). The patient was admitted for acute hypoxic respiratory failure secondary to COVID-19 and for management of infectious versus inflammatory colitis.

**Figure 3:**
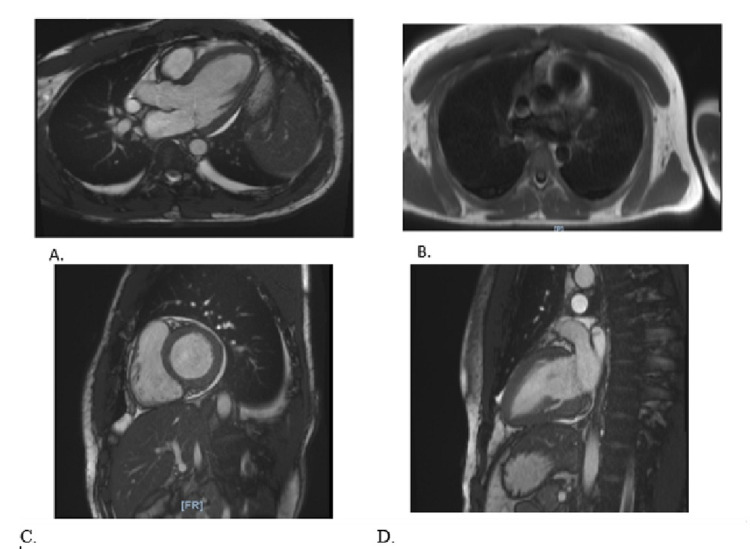
Cardiac MRI with gadolinium-enhanced contrast. Cardiac MRI is performed with bright blood and black blood technique pre- and post-contrast administration. Dynamic contrast enhancement and delayed myocardial viability/scarring images are obtained (A-D). There is myocardial edema at the mid-lateral wall to the apex (C and D). There is global hypokinesia on the cine views is trace bilateral pleural effusion and pericardial effusion (A and B). Aortic valve is tricuspid and without elevation of peak systolic velocity or abnormality of area grossly evident. End-diastolic volume is below normal and the end-systolic volume is above normal. All of the parameters are within normal. Right ventricular ejection fraction 42% and stroke volume almost identical to the left.

The patient was treated with intravenous hydration, started on 2 liters nasal cannula (NC) supplemental oxygen, eventually requiring OxyMask (Southmedic Inc., Canada), dexamethasone 6 milligrams daily, and remdesivir for five days. He was started on vancomycin 1.25 grams every 12 hours and Zosyn 3.375 milligrams daily for the treatment of colitis. Although he was admitted for abdominal pain secondary to colitis and was found to be hypoxic due to COVID-19-positive infection, he underwent a transthoracic echocardiogram due to the abnormal EKG and troponins on admission. There were incidental findings of decreased left ventricular ejection fraction to 50-55%, mild inferior septal and inferior wall hypokinesis, and a trivial pericardial effusion. Due to the patient’s echocardiogram findings and his elevated brain natriuretic peptide (BNP) and troponin, myocarditis was on the differential. The patient underwent cardiac magnetic resonance imaging (MRI), which revealed diminished biventricular ejection fraction with mild global hypokinesis, lateral apical wall myocardial edema, and epicardial enhancement (Figures 3A-3D). These findings were consistent with myocarditis. Further workup revealed positive high titers of Coxsackie B1-B6 antibodies. Infectious disease was consulted and suggested that either etiology (COVID-19 or Coxsackievirus) could be viable for the given clinical presentation. Following cardiac MRI, the next step in workup would be endomyocardial biopsy to reliably establish the cause. The patient’s hospitalization was also complicated by persistent atrial fibrillation and was unsuccessfully treated with numerous attempts at synchronized cardioversion. Two hours after unsuccessful cardioversions, the patient spontaneously converted to normal sinus rhythm but was maintained on sotalol for three days for rhythm control. The patient remained hospitalized for a total of 10 days and although hemodynamically stable continued to experience exercise intolerance and fatigue. He was medically optimized and discharged home on two more days of dexamethasone, to complete a total 10-day course of steroids. Unfortunately, the patient moved to another state to follow up with physicians closer to his hometown.

## Discussion

Clinical presentation of myocarditis can be variable and is dependent on the underlying etiology [1,8]. Patients may present with chest pain, fever, dyspnea, arrhythmias, and signs of congestive heart failure [1,8]. An increase in cardiac enzymes (troponins, creatine-kinase-MB) without any underlying coronary pathology can also be seen [1,8]. Myocarditis is a major cause of sudden cardiac death, especially in adults younger than 40 years of age [1,6,8]. Other notable complications include heart block, dilated cardiomyopathy, and thrombus with systemic emboli [1,6,8]. Although our patient had common complications including cardiac arrhythmias, this case is also unique as he presented with colitis, a symptom not typically seen in myocarditis.

It is known that Coxsackievirus is a cause of myocarditis; however, recent studies have shown increasing prevalence of COVID-19 induced myocarditis [1,2,8]. Our patient had multiple etiologies of myocarditis, including the COVID-19 infection, COVID-19 vaccination, and the Coxsackievirus. In viral myocarditis, symptoms develop weeks after the initial viral exposure, which means that any of these etiologies were plausible differentials [2,3,7]. Thus, it was difficult to determine the exact cause of myocarditis in this scenario. As the patient was COVID-19 positive, it was initially presumed that COVID-19 was the cause of myocarditis. However, as the patient had high titers of Coxsackie B1-B6 antibodies, this too may have contributed to the development of myocarditis. Although previous studies have shown COVID-19 myocarditis to be very common, those studies have not included patients with concurrent viral illnesses and moreover have not been diagnosed with COVID-19 infection after a complete vaccination series [5,9-11]. This complicates the confidence in diagnosing COVID-19 as the cause of myocarditis in this patient [5,9-11].

While currently nonspecific inflammatory cardiac markers, EKG, echocardiography, and cardiac MRI have been utilized for diagnostic and prognostic value for myocarditis; there is a need for immediate action to prevent cardiac failure secondary to fulminant myocarditis [4]. Histology of an endomyocardial biopsy is required to diagnose myocarditis and its etiology, but the utility of the biopsy is limited because of the sampling error due to the patchy inflammation of the myocardium [2,3]. Additionally, biopsies are invasive and not commonly performed because Myocarditis is typically treated with steroids and anti-arrhythmic agents regardless of etiology. Although we did not biopsy the myocardium, lysis of the myocardial cells or notable T lymphocyte infiltration within the biopsy could have distinguished the etiology [3]. Other less utilized tests include acute and chronic convalescent plasma titer levels.

## Conclusions

SARS-CoV-2 has been shown to cause multiple organ system complications; however, long-term prognosticators have not been studied extensively. Understanding the etiology could prove effective in aiding prognostication, providing a better framework for tailoring therapy as controlling progression is paramount given the limited literature on cardiac sequelae of COVID-19. Further research in treatment protocol for myocardial injury secondary to COVID-19 is required for a better approach to future myocarditis cases. This case is quite complex due to the numerous overlapping etiologies, which include active COVID-19 infection, recent Moderna COVID-19 vaccination series, or the Coxsackievirus. Understanding the underlying etiology is important for long-term prognosticators and short-term treatment in patients with myocarditis. Thus, it is paramount that appropriate workup should be completed for patients with unclear etiologies.
